# Growth Hormone Promotes Motor Function after Experimental Stroke and Enhances Recovery-Promoting Mechanisms within the Peri-Infarct Area

**DOI:** 10.3390/ijms21020606

**Published:** 2020-01-17

**Authors:** Sonia Sanchez-Bezanilla, N. David Åberg, Patricia Crock, Frederick R. Walker, Michael Nilsson, Jörgen Isgaard, Lin Kooi Ong

**Affiliations:** 1 School of Biomedical Sciences and Pharmacy and the Priority Research Centre for Stroke and Brain Injury, the University of Newcastle, University Dr, Callaghan, NSW 2308, Australia; sonia.sanchezbezanilla@uon.edu.au (S.S.-B.); rohan.walker@newcastle.edu.au (F.R.W.); michael.nilsson@newcastle.edu.au (M.N.); 2 Brain and Mental Health, Hunter Medical Research Institute, Lot 1, Kookaburra Cct, New Lambton Heights, NSW 2305, Australia; patricia.crock@newcastle.edu.au; 3 Department of Internal Medicine, University of Gothenburg, 405 30 Gothenburg, Sweden; david.aberg@medic.gu.se; 4 Department of Internal Medicine, Region Västra Götaland, Sahlgrenska University Hospital, Blå stråket 5, 413 45 Gothenburg, Sweden; 5 Department of Paediatric Endocrinology and Diabetes, John Hunter Children’s Hospital, Kookaburra Cct, New Lambton Heights, NSW 2305, Australia; 6 NHMRC Centre of Research Excellence Stroke Rehabilitation and Brain Recovery, 245 Burgundy Street, Heidelberg, VIC 3084, Australia; 7 Centre for Rehab Innovations, Lot 1, Kookaburra Cct, New Lambton Heights, NSW 2305, Australia; 8 LKC School of Medicine, Nanyang Technological University, 50 Nanyang Ave, Singapore 639798, Singapore; 9 School of Pharmacy, Monash University Malaysia, Bandar Sunway, Subang Jaya 47500, Selangor, Malaysia

**Keywords:** Ischemic stroke, growth hormone, motor recovery, neurogenesis, neuronal plasticity, vascular remodelling

## Abstract

Motor impairment is the most common and widely recognised clinical outcome after stroke. Current clinical practice in stroke rehabilitation focuses mainly on physical therapy, with no pharmacological intervention approved to facilitate functional recovery. Several studies have documented positive effects of growth hormone (GH) on cognitive function after stroke, but surprisingly, the effects on motor function remain unclear. In this study, photothrombotic occlusion targeting the motor and sensory cortex was induced in adult male mice. Two days post-stroke, mice were administered with recombinant human GH or saline, continuing for 28 days, followed by evaluation of motor function. Three days after initiation of the treatment, bromodeoxyuridine was administered for subsequent assessment of cell proliferation. Known neurorestorative processes within the peri-infarct area were evaluated by histological and biochemical analyses at 30 days post-stroke. This study demonstrated that GH treatment improves motor function after stroke by 50%–60%, as assessed using the cylinder and grid walk tests. Furthermore, the observed functional improvements occurred in parallel with a reduction in brain tissue loss, as well as increased cell proliferation, neurogenesis, increased synaptic plasticity and angiogenesis within the peri-infarct area. These findings provide new evidence about the potential therapeutic effects of GH in stroke recovery.

## 1. Introduction

Globally, stroke is a leading cause of disability [1]. The most common and widely recognised neurological impairments caused by stroke are deficits in motor function [2]. Therefore, motor-based rehabilitation techniques have been developed to promote the recovery of motor impairments in stroke patients [3,4,5,6,7]. Current clinical practice for impaired sensorimotor functions are mainly based on physical therapy starting within 48 h of stroke onset, which can last for as long as months and years after the stroke [4,8]. Rehabilitation after stroke is often a long and slow process, and therefore, the development of new effective therapeutic strategies that can enhance the recovery of brain function and improve functional outcomes are highly desirable. An innovative solution may be the use of a pharmacological strategy to promote a pro-restorative environment within the brain. One promising strategy in this regard is growth hormone (GH). 

The therapeutic potential of GH on brain repair after brain injury or stroke has been considered in both human [9,10,11,12,13,14,15,16,17,18] and preclinical [19,20,21,22,23,24] studies. GH, a peptide hormone released from the anterior pituitary gland, plays an important role in brain growth, development and function [11]. Critically, prior work has documented the widespread expression of GH and growth hormone receptor (GHR) in the rat brain [25], and the ability of GH to stimulate the genesis of neuronal stem cells [26] and endothelial cells, as well as to promote synaptogenesis [27]. It is well known that any injury to the adult brain generates an adaptive brain repair response, which includes the proliferation of new cells and differentiation into different populations [28,29,30,31]. Previous experimental studies have shown that ischemic injury induces proliferation of newly born neurons in the subventricular zone, migration of these immature neurons and localization within the peri-infarct region [31,32,33,34,35,36]. However, this self-repair mechanism operates only acutely after stroke and is insufficient to promote long-term recovery. Interestingly, several experimental studies have shown that GH can promote these neurorestorative or plasticity-promoting processes beyond what occurs with spontaneous recovery after brain injury or stroke, and this increased neurorestoration is closely linked with improvement in functional outcomes [9,11,15,18,37,38]. Despite the fact that the positive effects of GH in the adult brain have been extensively studied, it is still not clear whether GH could promote motor function after stroke.

In previous studies, we assessed the pro-cognitive effects of peripherally administered GH after experimental stroke and the possible underlying mechanisms leading to such effects. We found that GH treatment promotes cognitive recovery after stroke, however sensorimotor effects were not evaluated. In addition, we found an increased expression of neurotrophic factors, markers of synaptogenesis and myelination, and the formation of new blood vessels [39]. However, there is still a large gap in our understanding of whether GH treatment could also enhance motor function after stroke, and whether this is associated with neurorestorative processes within the peri-infarct regions.

In this study, we aimed to evaluate whether GH treatment could promote motor function after experimental stroke. Our primary hypothesis was that mice who received stroke that were then treated with recombinant human growth hormone (rhGH) for 28 days, starting at 48 h post-stroke, would improve performance in motor tasks relative to non-rhGH-treated stroke mice. Our secondary hypothesis was that the rhGH-treated stroke mice would show an enhancement in known neurorestorative processes within the peri-infarct region. We assessed cellular and molecular changes using comprehensive and cross-validated approaches, including bromodeoxyuridine (BrdU) tagging, co-immunolabelling and western blotting analyses. 

## 2. Results

### 2.1. GH Treatment Improves Motor Function

**Figure 1:**
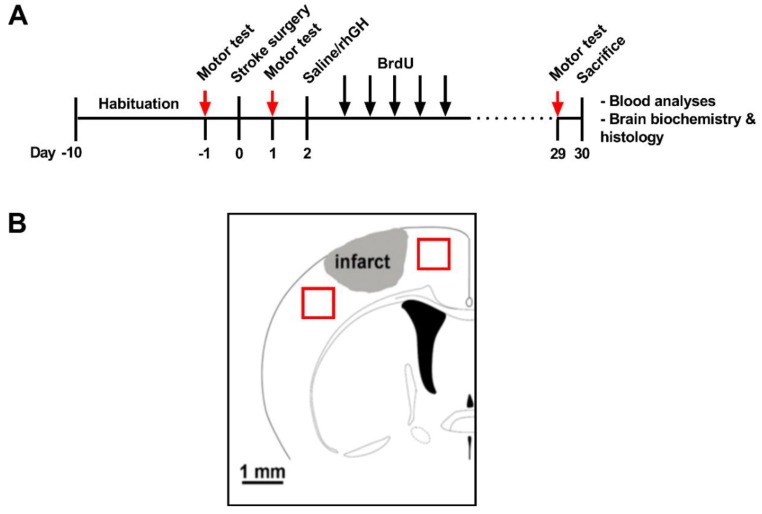
(**A**) Experimental timeline. Photothrombotic stroke was induced in all mice. Two days post-stroke, mice were randomly treated with either saline or recombinant human growth hormone (rhGH) via mini-osmotic pumps for 28 days. At day 3, mice were injected with bromodeoxyuridine (BrdU) for 5 consecutive days. Mice were assessed by motor tests at one day before stroke (pre-stroke), one day after stroke (post-stroke) and 29 days post-stroke (post-treatment). (**B**) Diagram illustrating the site of photothrombotic stroke induction (grey area) at Bregma 0.0 mm. Red squares represent the area of the peri-infarct region selected for immunofluorescence analyses. The peri-infarct territory, which is found in the 2 mm around the infarct core, was dissected for protein analysis. Bar = 1 mm.

**Figure 2:**
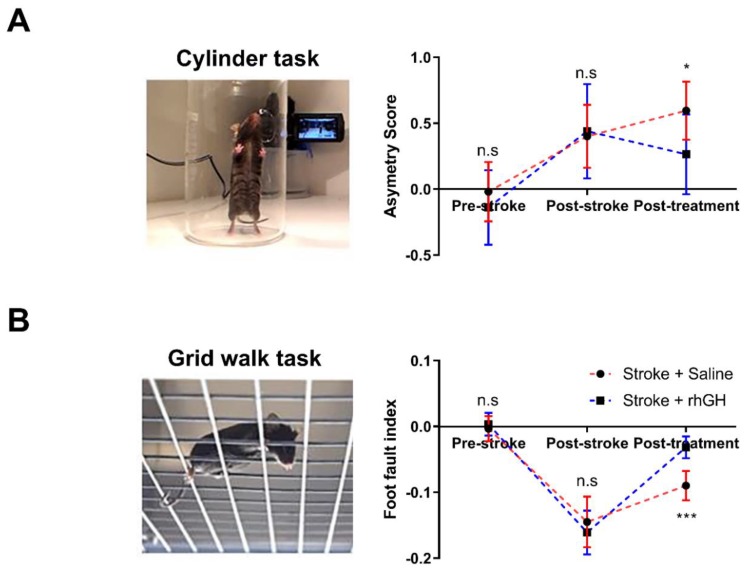
The effects of recombinant human growth factor (rhGH) treatment post-stroke on motor function. (**A**) Asymmetry scores were evaluated by the cylinder test, which shows that mice that received rhGH treatment significantly improve motor function. (**B**) A foot fault index was evaluated by the grid walk test, which also shows an improvement in motor function after rhGH treatment. In all panels, red colour designates saline treatment and blue designates rhGH treatment. Mean ± standard deviation (SD). n.s = not significant. * *p* < 0.05 and *** *p* < 0.001.

We treated the mice with either saline (*n* = 10) or rhGH (*n* = 10) using a mini-osmotic pump, starting at 48 h post-stroke for 28 days. Mice were assessed for motor deficits one day before stroke (pre-stroke), one day after stroke (post-stroke) and at 29 days post-stroke (post-treatment) (Figure 1A). We used the cylinder task to evaluate locomotor asymmetry. This task evaluates the forelimb preference that mice utilise for upright postural support when rearing up on the cylinder wall. Data on the asymmetry scores indicated that there were no significant differences in forelimb preference prior to stroke. One day after stroke, all mice showed a significantly stronger preference for using their ipsilateral (unaffected) forelimb. At 29 days post-stroke, we found a significant motor improvement (50.32%, *p* = 0.0123) in rhGH-treated stroke mice compared with saline-treated mice (Figure 2A). Motor function was also assessed using the grid walk task. This task evaluates the ability of mice to effectively place their paws on an elevated grid during locomotion. As expected, there was no difference in the number of foot faults before stroke. One day after stroke, the number of foot faults on the contralateral (affected side) was significantly higher in all stroke mice. At 29 days post-stroke, there was a significant effect of rhGH treatment on motor function recovery (64.27%, *p* < 0.0001; Figure 2B).

### 2.2. GH Treatment Increases Plasma IGF-1 Levels 

rhGH treatment administered post-stroke significantly increased insulin-like growth factor 1 (IGF-1) (Saline treatment 289.9 ± 36.14 versus rhGH treatment 433.5 ± 44.2 ng/mL, 49.51%, *p* < 0.0001) and IGFBP-3 (Saline treatment 254.3 ± 50.06 versus rhGH treatment 293.7 ± 26.1 ng/mL, 15.49%, *p* = 0.0405) levels in plasma, assessed at time of sacrifice. These results have confirmed that commercially available rhGH has significant effects on mouse physiology when delivered subcutaneously via a mini-osmotic pump. Further, we assessed the association between plasma IGF-1 and motor performance. A Pearson correlation analysis showed a significant positive correlation between plasma IGF-1 levels and cylinder task performance post rhGH treatment (r = −0.6789; *p*_(Y = –0.002513X + 1.339)_ = 0.0010) (Supplementary Figure S3A). There was also a significant correlation between plasma IGF-1 levels and the grid walk task performance post rhGH treatment (r = 0.8879; *p*_(Y = 0.0003785X – 0.1977)_ < 0.0001) (Supplementary Figure S3B).

### 2.3. GH Treatment Reduces Tissue Loss

**Figure 3:**
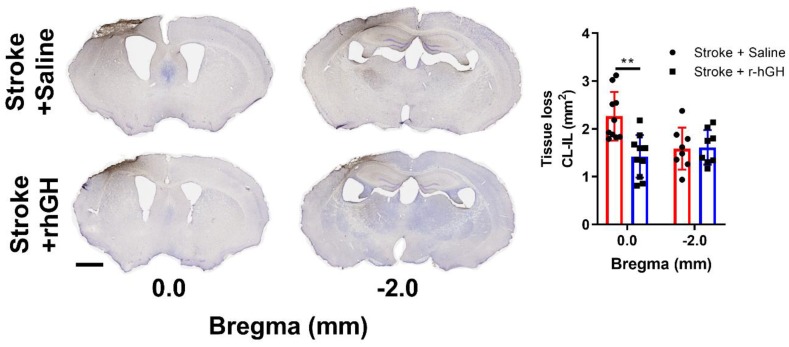
Cresyl violet staining of brain sections from Bregma 0.0 and –2.0 mm. Tissue loss was calculated as contralateral (CL) hemisphere area − ipsilateral (IL) hemisphere area (mm^2^). ** *p* < 0.01. Bar = 1 mm.

Using Cresyl Violet staining, we estimated the tissue loss at 0.0 and –2.0 mm from Bregma. We found a significant decrease in tissue loss at Bregma 0.0 mm at 30 days post stroke in mice treated with rhGH (29.89%, *p* = 0.0088; Figure 3).

### 2.4. GH Treatment Promotes Cell Proliferation and Neurogenesis within the Peri-Infarct Region

**Figure 4:**
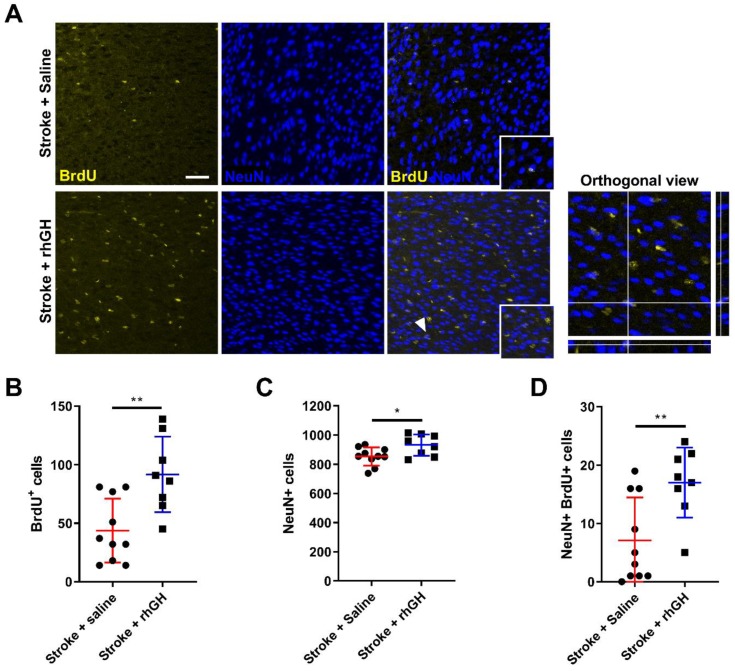
rhGH treatment promotes cell proliferation and neurogenesis in the peri-infarct region. (**A**) Representative immunofluorescence images, which are co-labelled with BrdU (yellow) and neuronal nuclei (NeuN, blue), with orthogonal view also presented (far right). The white arrowhead shows a co-labelled cell visualised in orthogonal view. (Scale bar = 50 μm). rhGH treatment post-stroke significantly increased the number of (**B**) BrdU-positive cells, (**C**) NeuN-positive cells, and (**D**) BrdU-NeuN-positive cells. Mean ± SD. * *p* < 0.05 and ** *p* < 0.01.

We assessed several selected neurorestorative effects of rhGH after stroke using an immunolabelling approach. Firstly, we performed BrdU and neuronal nuclei (NeuN) co-labelling (Figure 4A). BrdU is an analogue of the nucleoside thymidine, which can be incorporated into replicating DNA and therefore can be used to identify proliferating cells [40]. In the peri-infarct region, we found a significant increase in the number of BrdU-positive cells in stroke mice treated with rhGH compared with saline-treated mice (109.67%, *p* = 0.0035; Figure 4B). We also observed an increase in the number of NeuN-positive neurons (9.10%, *p* = 0.0256) (Figure 4C). To assess whether these proliferating cells have differentiated into neurons, we analysed co-localisation of BrdU and NeuN labelling. rhGH treatment post-stroke significantly increased the number of BrdU-NeuN-positive cells (139.44%, *p* = 0.0073; Figure 4D).

**Figure 5:**
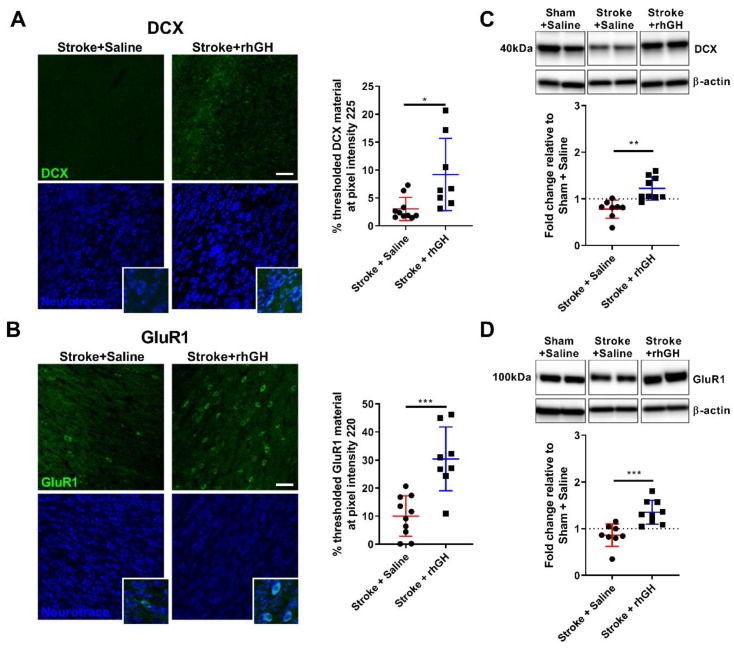
Increased expression of both Doublecortin (DCX) and α-amino-3-hydroxy-5-methyl-4-isoxazolepropionic acid receptor 1 (GluR1) in the peri-infarct region after rhGH treatment. (**A**) Representative confocal images of peri-infarct areas co-labelled with DCX (green) and Neurotrace (blue) and high magnification (Scale bar = 50 μm). Quantification of material threshold at the pixel intensity 225 shows an increase in DCX-positive structures after rhGH treatment. (**B**) Representative confocal images of peri-infarct areas co-labelled with GluR1 (green) and Neurotrace (blue), and high magnification (Scale bar = 50 μm). Quantification of material threshold at the pixel intensity 220 shows increased GluR1-positive structures after rhGH treatment. (**C**) Representative western blot and quantification using anti-DCX antibody within the peri-infarct region. Quantification revealed increased expression of DCX in rhGH-treated mice. (**D**) Representative western blot and quantification using anti-GluR1 antibody within the peri-infarct region. Quantification revealed increased expression of GluR1 in rhGH-treated mice. Mean ± SD. * *p* < 0.05, ** *p* < 0.01 and *** *p* < 0.001.

Next, we analysed the levels of doublecortin (DCX), a marker of immature neurons [41]. DCX is a microtubule-associated protein, which is necessary in the proliferation of progenitor cells during neurogenesis [42,43]. Optical density of DCX immunofluorescence images within the peri-infarct region were quantitatively assessed using a threshold analysis protocol. This immunofluorescence data revealed a significant increase in material thresholded for DCX (201.97%, *p* = 0.0115, at pixel intensity 225; Figure 5A) in stroke mice treated with rhGH compared with saline treatment. We further confirmed this histology data using a western blot protocol. The protein homogenates from the peri-infarct region of stroke + saline and stroke + rhGH cohorts were analysed along with a sham + saline cohort. We found a significant increase in DCX levels (0.44-fold, *p* = 0.0010; Figure 5C) after rhGH treatment.

### 2.5. GH Treatment Promotes Expression of GluR1 within the Peri-Infarct Region

We also assessed the levels of α-amino-3-hydroxy-5-methyl-4-isoxazolepropionic acid receptor 1 (GluR1). GluR1 is a receptor implicated in synapse formation, stabilisation, and plasticity [44]. The immunofluorescence data revealed a significant increase in material threshold for GluR1 (203.54%, *p* = 0.0003, at pixel intensity 220; Figure 5B) in stroke mice treated with rhGH compared with saline-treated mice. The western blot data also shows a significant increase in GluR1 (0.49-fold, *p* = 0.001; Figure 5D) levels after rhGH treatment. 

### 2.6. GH Treatment Promotes Angiogenesis within the Peri-Infarct Region

**Figure 6:**
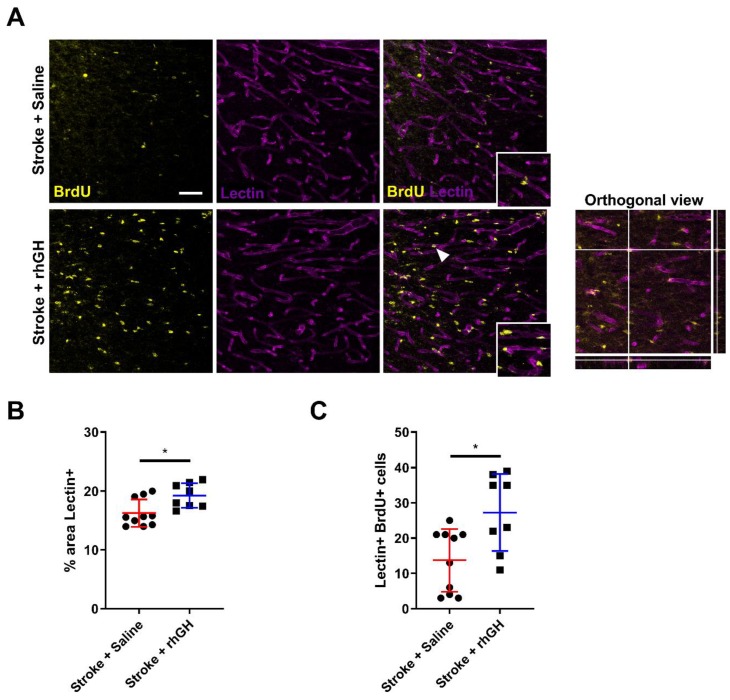
rhGH treatment promotes angiogenesis in the peri-infarct region post-stroke. (**A**) Representative immunofluorescence images of cells co-labelled with BrdU (yellow) and Lectin (purple) and orthogonal view (far right). The white arrowhead shows a cell visualised in orthogonal view. (Scale bar = 50 μm). rhGH treatment post-stroke significantly increased the % of area that was positive for (**B**) Lectin and (**C**) the number of BrdU-Lectin-positive cells. Mean ± SD. * *p* < 0.05.

We next analysed the brain vasculature after rhGH treatment (Figure 6A). To assess vascular density, we used Lectin as a blood vessel staining and we performed a digital reconstruction of vessels for analysis of the immunofluorescence images. We found a significant increase in the percentage area covered by Lectin within the peri-infarct region in stroke mice treated with rhGH compared with saline treatment (18.20%, *p* = 0.0129; Figure 6B). 

To assess whether the BrdU-positive cells have differentiated into endothelial cells, we performed co-labelling analyses of BrdU with Lectin. We found that rhGH treatment post-stroke significantly increased the number BrdU-Lectin-positive cells (98.91%, *p* = 0.0103; Figure 6C).

## 3. Discussion

The main finding of the present study is that GH treatment improves motor function after experimental stroke. Specifically, GH treatment for 28 days starting 48 h post photothrombotic stroke significantly improved motor function, when compared with non-GH-treated stroke mice. Motor function was assessed using two different tasks, and in both tasks, GH treatment significantly improved motor deficits. GH treatment reduced the preference for spontaneous use of ipsilateral forelimbs during the cylinder task and decreased the number of foot-faults on the grid walk task. Secondly, we studied selected neurorestorative processes, which have been previously linked to motor improvement. We observed an increase in proliferation of progenitor cells, neurogenesis, increased synaptic plasticity and angiogenesis within the peri-infarct area. Collectively, these results provide novel evidence supporting the use of GH after stroke to enhance known neurorestorative processes within the peri-infarct region, leading to an improvement in motor function. 

In our previous study, we demonstrated that GH treatment post-stroke stimulates cognitive recovery in mice [39]. However, we did not investigate whether GH could improve motor function after stroke. Currently, therapies that promote functional motor recovery after stroke are exclusively limited to physical rehabilitation and secondary prevention, with only a modest degree of recovery [45]. While rehabilitation plays an important role in recovery from stroke, a pharmacological therapy to enhance this recovery would be highly desirable. GH is a particularly interesting option due to its approval profile, efficacy and safety. Furthermore, there is now building evidence demonstrating that GH treatment in addition to standard rehabilitation can significantly contribute to the motor recovery of an acquired brain injury [9,10,11,12,13,14,15,16,17,18,19,20,21,22,23,24,37,46,47]. For instance, Heredia et al. [18,37] observed that administration of GH subcutaneously together with rehabilitation significantly improved motor function in rats as measured by the paw-reaching-for-food task. Therefore, we were motivated to extend the existing preclinical literature to consider GH treatment for its ability to ameliorate motor impairments after stroke [14].

In the present study, we aimed to investigate the effect of GH alone on motor function using a photothrombotic stroke model. Specifically, we utilised a battery of tests to assess the impact of GH on motor function. The cylinder and grid walk tests are standard motor function assessments, which have been extensively validated and used in experimental stroke studies [48]. As expected, photothrombotic vascular occlusion of the motor and somatosensory cortices resulted in a clear impairment of motor function at 1-day post-stroke, which persisted for up to 30 days. The stroke mice showed preferential use of the unaffected forelimb and an increase in foot faults when using the impaired limb(s). Critically, GH treatment had a significant effect on enhancing recovery from these motor deficits at 30 days post-stroke, ameliorating the preferential use of the unaffected limb and reduction in the number of foot fault errors, thus confirming our primary hypothesis. It should be noted that our findings differ from the results from a study conducted by Pathipati et al. [14]. Although they found minor positive effects on motor recovery as assessed by a forepaw inhibition test, they did not observe any significant difference in forelimb asymmetry (cylinder task) after stroke between the GH and vehicle treatment groups. This discrepancy may be accounted for by different experimental stroke models (photothrombotic versus middle cerebral artery occlusion) and GH delivery (subcutaneous versus intracerebroventricular). In addition, we identified that GH treatment enhanced circulating levels of IGF-1 and its primary circulating binding protein, IGFBP-3. Previous studies have demonstrated the effect of IGF-1, a primary mediator of GH, on improving motor function after stroke [49,50]. Our data also suggests that higher circulatory levels of IGF-1 are associated with better motor outcomes. 

To extend our understanding of the mechanisms behind the positive effects of GH on motor function, we also examined the impact of GH in known neurorestorative processes. Specifically, we focused on proliferation of progenitor cells, neurogenesis, synaptic plasticity and cerebrovascular remodelling. We observed a significant reduction in the tissue loss from the ipsilateral hemisphere at Bregma 0.0 mm in stroke mice treated with GH, which is consistent with our previous observation [39]. To interpret these results, we have to consider that in this study, we started the GH treatment at 48 h post-stroke. At this time point, most of the neurons within the infarct would have died [51] and therefore, we would not link the decrease in tissue loss to a neuroprotective effect of GH. Instead, we would suggest that the reduction of tissue loss of the ipsilateral hemisphere is attributed to an increase in neurorestorative processes. To support our hypothesis, we used BrdU tagging to assess proliferating cells. We observed an increased number of BrdU-positive cells within the peri-infarct regions in stroke mice treated with GH, which is consistent with previous studies demonstrating that GH promotes cell proliferation within the central nervous system [52,53]. Critically, these progenitor cells are known to release neurotrophic factors to provide an environment which may contribute to neural network remodelling and functional recovery [54,55].

We then studied the fate of these newly proliferating cells. Firstly, we analysed the number of BrdU-NeuN-positive cells and we observed an increase in the number of these cells in stroke mice treated with GH. We would interpret that the new proliferating cells have differentiated (or matured) into neurons, supporting the idea that GH promotes neurogenesis after stroke [56]. Secondly, we considered the levels of expression of DCX, a protein mainly expressed by immature neurons in neurogenic niches [57,58,59]. Interestingly, previous studies have demonstrated that the number of DCX-positive cells positively correlates with recovery from functional deficits after stroke and, on the other hand, conditional ablation of DCX deteriorates both short- and long-term functional outcomes post-stroke [60,61,62]. Specifically, Jin et al. [61] showed that depletion of DCX exacerbated sensorimotor behavioural deficits measured by rotarod, limb placing and elevated body swing tests. Here, we showed that GH treatment in stroke mice also resulted in a significant increase in DCX-positive structures and protein levels within the peri-infarct region. The ability for GH to promote neurogenesis within the peri-infarct region after stroke is a critical finding, as previous studies reported an association between functional motor recovery and the number of newly born neurons in the motor and somatosensory cortex after ischemic injury [63].

Synaptic plasticity is also known to play a critical role in recovery post-stroke [64]. We investigated the AMPA receptor subunit GluR1, which is implicated in synapse formation, stabilisation and plasticity [65,66,67]. Previous studies have indicated that an enhancement in synaptic plasticity, specifically AMPA signalling, promotes motor recovery after stroke [68]. Clarkson et al. [68] showed that positive allosteric modulators of AMPA receptors enhance motor recovery when administered after stroke, while AMPA receptor antagonists impair motor recovery in a photothrombotic stroke model. Here, we identified an increase in GluR1-positive structures and protein levels within the peri-infarct region post-stroke in the GH-treated group. This finding further supports that GH plays an important role in synaptogenesis after stroke, as we previously observed an increase in the protein levels and density of synapsin-1 after GH treatment in a stroke model [39]. 

Post-stroke angiogenesis is an essential process to restore brain function, leading to functional recovery [69]. Over several weeks after stroke, spontaneous proliferation of capillary endothelial cells and a gradual increase of revascularisation had been observed [70,71,72]. Furthermore, most of the neurorestorative agents that improve functional outcomes after stroke or brain injury increase angiogenesis [73,74,75,76,77]. For instance, Chen et al. demonstrated that statins effectively enhanced motor function and this beneficial effect appeared to be mediated by an increase in angiogenesis, neurogenesis and synaptogenesis [76,78]. In addition, GH has been previously associated with angiogenesis in various areas of the brain. Sonntag et al. demonstrated that GH treatment increases cerebrocortical arteriolar density in aged rats [79]. It is therefore reasonable to propose that angiogenesis might be upregulated in the brain after GH treatment and may contribute to the improvement in motor function. Here, we analysed vessel density in the peri-infarct area using immunofluorescence labelled tomato-Lectin, which binds to glycoproteins located in the glycocalyx and in the basal membrane of endothelial cells [80]. Our results indicate that GH increases vessel density and area coverage at 30 days post-stroke. Critically, we observed an increase in the number of BrdU-Lectin-positive cells in the mice treated with GH following stroke, suggesting that GH has the ability to enhance angiogenesis. These findings align with our previous study, where we showed that GH has a positive effect on cerebrovascular remodelling by increased density and area coverage of both cluster of differentiation 31 (CD31) and collagen-IV-positive cells, which are typical endothelial cell markers commonly used to identify blood vessels. 

One potential limitation of this study is that we did not consider the effect of GH on inflammatory processes after stroke. It is well documented that neuroinflammation also plays an important role in the pathophysiology of acute brain ischemia. Such phenomena are characterised by rapid activation of microglia, astrogliosis, infiltration of peripheral immune cells and production of pro-inflammatory cytokines [81,82,83,84,85,86,87]. Interestingly, GH has been previously demonstrated to induce gliogenesis [11]. It would certainly be worthwhile to investigate the effect of GH on neuroinflammatory processes after stroke in future studies.

In conclusion, in this study we demonstrated that peripheral GH treatment improves motor function post-stroke. This motor improvement is associated with enhancement of neurorestorative processes such as cell proliferation, neurogenesis, synaptic plasticity and angiogenesis. Collectively, our results reinforce the concept of using GH as a useful therapeutic tool in promoting brain recovery post-stroke. This may be clinically relevant as there are studies documenting high incidence of GH dysregulation after stroke [88,89,90]. While our results are encouraging and support earlier promising results from our group and others, further research should consider how long after the initial stroke we could start the treatment, what would be the optimal dose and timing required to promote functional recovery and how long after the cessation of the treatment do the positive outcomes persist, as well as the interaction of the treatment with common comorbidities. Further, the usage of GH as an adjuvant during rehabilitation after stroke should be considered. We propose that GH appears to represent a promising therapeutic intervention after stroke and should be considered for clinical studies. 

## 4. Materials and Methods

### 4.1. Animals 

Male C57BL/6 mice (10 weeks; *n* = 48) were provided by the Animal Services Unit at the University of Newcastle (Newcastle, New South Wales, Australia). Mice were housed in cages with food and water available ad libitum in a temperature- (21 °C ± 1) and humidity-controlled environment. The room lighting was set on a 12:12 h reverse light–dark cycle with lights on at 19:00. All animal procedures were conducted during the dark phase. Prior to the initiation of the experiments, mice were allowed to acclimatise to the environment for seven days. Experiments were approved by the University of Newcastle Animal Care and Ethics Committee (A-2014-432, 23 November 2015) and conducted in accordance with the New South Wales Animals Research Act and the Australian Code of Practice for the use of animals for scientific purposes. The ARRIVE guidelines (Animal Research: Reporting of In Vivo Experiments) were adhered for all animal procedures. Mice were randomised into the experimental groups. Behavioural assessments, histological and biochemistry analyses were performed in a blinded manner (see Supplementary Figure S1 for details about animal number and inclusion/exclusion criteria). 

### 4.2. Sample Size Calculation

Sample size was estimated using G*Power 3.1 software (Heinrich Heine University Düsseldorf, Düsseldorf, Germany). Calculations were made based on previous data on rhGH treatment after experimental stroke [39], we obtained an effect size of d = 1.6. Allowing a type 1 error of 5%, α = 0.05, with the power of 80%, β = 0.2, we calculated a sample size of 8 animals per group. 

### 4.3. Experimental Design

The first cohort of mice (*n* = 24) was used to investigate the neurorestorative effects of rhGH in promoting motor function recovery, and to explore the underlying mechanisms using histological analysis (see Figure 1A for timeline). On Day 0, all mice underwent photothrombotic occlusion. Treatment with rhGH (1.4 mg/kg body weight per day) or saline began on Day 2. rhGH was delivered subcutaneously via mini-osmotic pumps and continued for 28 days. At day 3 post-stroke, mice were injected with BrdU (50 mg/kg body weight/day, Sigma-Aldrich, St. Louis, MO, USA), continuing for 5 consecutive days. Motor function was assessed one day prior to stroke (pre-stroke), Day 2 (post-stroke) and at 29 days post-stroke (post-treatment). On day 30, mice were euthanised using sodium pentabarbitol. Plasma samples were collected for analysis of insulin-like growth factor 1 (IGF-1) and insulin-like growth factor-binding protein 3 (IGFBP-3) levels using enzyme-linked immunosorbent assay (ELISA) kits (R&D systems, Minneapolis, MN, USA). After blood was collected, mice were perfused, and brains were collected for histological analysis.

The second cohort of mice (*n* = 24) was generated for protein biochemical analysis of the peri-infarct territory. On Day 0, all mice were randomly allocated to receive either photothrombotic occlusion or sham surgery. Treatment with rhGH (1.4 mg/kg body weight per day, or saline control) or saline began on Day 2, subcutaneously via mini-osmotic pumps for 28 days. Mice were sacrificed on day 30 and brains were collected for biochemical analysis.

### 4.4. Photothrombotic Occlusion 

Photothrombotic occlusion was performed as described previously [91,92,93]. Firstly, mice were anesthetized with 2% isoflurane and placed on a temperature-controlled (37 °C ± 1) stereotaxic frame. Mice received an intraperitoneal injection of Rose Bengal (200 μL, 10 mg/mL solution in sterile saline, Sigma-Aldrich, USA) or sterile saline (0.9% NaCl, Pfizer, Sydney, Australia) before the skull was exposed by incision of the skin. At 8 min post-injection, a cold light source (4.5 mm diameter) was placed on the skull at 2.2 mm left lateral of Bregma 0.0 mm for 15 min.

### 4.5. Mini-Osmotic Pump Placement 

Mini-osmotic pump placement was performed as previously described [94]. Briefly, at 48 h post-stroke, mice were anaesthetised with 2% isoflurane. An incision was made in the skin between the scapulae to create a subcutaneous space for the mini-osmotic pumps (Model 2004, Alzet, Cupertino, CA, USA). The mini-osmotic pumps were filled with 200 μL of either rhGH (Somatropin 10 mg/1.5 mL, SciTropin A, SciGen, Australia) or sterile saline. The pumps deliver 0.25 μL/hour for 28 days (0.04 mg rhGH per day). After the pump was placed into the subcutaneous space, the skin incision was closed with staples. The surgical procedure was performed in a temperature-controlled environment (37 °C ± 1).

### 4.6. Motor Test

As outlined in the experimental design, motor tests were performed one day pre-stroke, one day post-stroke and at 29 days post-stroke.

Forelimb asymmetry was assessed by a cylinder test as previously described [86,92,95]. Briefly, each mouse was placed in a glass cylinder. The locomotor activity was recorded using video cameras from two different angles. The first forelimb to touch the wall of the cylinder during a full rear was scored as a wall placement. When both forelimbs (left and right) simultaneously touched the wall of the cylinder, it was considered as one placement for each forelimb. A total of 20 forelimb placements per mouse were scored by a blinded researcher. The forelimb asymmetry score was calculated as the ratio of non-impaired forelimb placement minus impaired forelimb placement to total forelimb placement. 

Deficits in limb placement were evaluated by the grid walk test as previously described [92,95]. Briefly, each mouse was placed on a grid (2 × 2 cm^2^) elevated from the ground. The locomotor activity was recorded using video cameras from two different angles. A ‘foot fault’ was scored when a mouse failed to place their paw on the bars of the grid. The number of foot faults on each side was counted over a total of 60 steps by a blinded researcher. A foot fault index was calculated as the ratio of non-impaired foot faults minus impaired foot faults to the total number of steps.

### 4.7. ELISA

Commercially available ELISA kits were used to measure plasma levels of IGF-1 (mouse/rat IGF-1 Quantikine ELISA (MG100; R&D systems, Minneapolis, MN, USA) and IGFBP-3 (mouse IGFBP-3 ELISA (EMIGFBP3; Thermo Fisher Scientific, Scoresby, Victoria, Australia), according to the manufacturer’s instructions. 

### 4.8. Histological Analysis

The first cohort of mice were perfused transcardially using 0.9% saline followed by 4% paraformaldehyde (pH 7.4, both solutions kept in ice). Brains were collected and post-fixed for 4 h in 4% paraformaldehyde. Brains were then transferred to a 12.5% sucrose solution in 0.1 M PBS and stored until sliced. Brains were sliced (coronal sections) using a freezing microtome (Leica, North Ryde, NSW, Australia) at a thickness of 30 μm and kept in an antifreeze solution at 4 °C. Fixed brains were later used for histological analyses.

Cresyl violet staining was performed as previously described [86]. Coronal brain sections (2 sections per brain, Bregma 0.0 and –2.0 mm) were mounted on glass slides and allowed to dry for 1 h. Sections were defatted in chloroform: consisting of submersion in ethanol solution for 8 min, followed by rehydration in absolute ethanol, 95% ethanol and 70% ethanol. Sections were stained in Cresyl Violet solution for 15 min. Then, sections were washed in 70% ethanol, 95% ethanol, differentiating solution and absolute ethanol. Finally, the sections were cleared in xylene and cover slipped.

Immunofluorescence staining was performed as previously described [85,96]. Free-floating fixed sections were rinsed in PBS, and 3% bovine serum albumin was used to block non-specific binding. For BrdU staining, antigen retrieval was performed before the blocking step, as follows: 10 min HCl (1 M) incubation on ice, 10 min HCl (2 M) incubation at room temperature, 20 min HCl (2 M) incubation at 37 °C, 10 min borate buffer (0.1 M) incubation at room temperature and three washes in PBS + 0.1% triton. Sections were incubated with the appropriate primary antibody (DCX, GluR1, BrdU, NeuN, (see Table 1) overnight at 4 °C. After the primary antibody incubation, sections were washed and incubated in corresponding secondary antibodies for 2 h at room temperature (see Table 1 for antibody concentrations and acronym description). For blood vessel detection, lectin staining was performed together with the secondary antibody incubation. Finally, brain sections were mounted on glass slides, air-dried and cover slipped.

### 4.9. Image Acquisition and Analysis

Cresyl Violet images were acquired at 20× magnification using Aperio AT2 (Leica, Wetzlar, Germany). The estimated tissue loss area was determined as the area of contralateral hemisphere – area of ipsilateral hemisphere using ImageJ software (National Institutes of Health, Bethesda, MD, USA). Bregma levels of 0.0 and –2.0 mm were used for analyses. 

Co-immunofluorescence high-resolution images of the peri-infarct area (Figure 1B) were taken on a Leica TCS SP8 confocal microscope with a Leica HC PLC APO 10×/0.40 objective. For the peri-infarct region of interest, 30 µm z-stacks with a step size of 1 µm were taken. Imaging parameters (laser power, resolution and gain) were held constant throughout all imaging sessions. Exhaustive automated BrdU and NeuN cell counts were performed using ImageJ software (Supplementary Figure S2A). For analysis of DCX and GluR1 labelling, we performed thresholding analyses and chose the optimal pixel intensity that clearly reflected the immunofluorescence signal (Supplementary Figure S2C). To measure vessel coverage (% Lectin-positive area), Lectin emission image was uniformly thresholded at a high stringency (Supplementary Figure S2B). The area of vessel coverage was expressed as a percentage of the overall image (ImageJ Software). For BrdU-NeuN-positive and BrdU-Lectin-positive co-labelling, we used the plugin ‘colocalization’ for ImageJ. The colocalised points of two 8-bits images were highlighted by this plugin and will appear as black points (Supplementary Figure S2D).

### 4.10. Protein Extraction and Western Blotting

The second cohort of mice were transcardially perfused with cold 0.9% saline for 3 min. Brains were dissected, snap frozen in isopentane and stored at −80 °C. A cryostat (−20 °C) was used to slice the brains (coronal sections) at a thickness of 200 μm. The peri-infarct territory (2 mm^2^ around infarct core, Bregma +1.0 to −1.0 mm) of each section was punched using a 1 mm tissue punch. Samples were stored in −80 °C for further biochemical analysis.

Protein extraction and subsequent western blotting were performed as previously described [91,97,98]. Briefly, peri-infarct tissue samples (Figure 1B) were sonicated in 300 μL lysis buffer. The components of the lysis buffer were 1% SDS, 1 mM ethylenediaminetetraacetic acid, 1 mM dithiothreitol, 80 μM ammoniummolybdate, 1 mM sodium pyrophosphate, 1 mM sodium vanadate, 5 mM β-glycerolphosphate, 1 protease inhibitor cocktail tablet, and 1 phosphatase inhibitor cocktail tablet in 50 mM tris(hydroxymethyl)aminomethane buffer pH 7.4. The samples were centrifuged at 14,000× *g* for 20 min at 4 °C. Then, supernatants were collected into new tubes to separate from the pellet fractions. For determination of protein concentration, a Pierce bicinchoninic acidprotein assay kit was used (Thermo Fisher Scientific, USA) per the manufacturer’s instructions. All supernatant samples were adjusted to 1.5 mg/mL. The samples were mixed with sample buffer (2% SDS, 50 mM Tris, 10% glycerol, 1% dithiothreitol, 0.1% bromophenol blue, pH 6.8). For western blotting, 15 μg of lysate was loaded per lane and electrophoresed to Biorad Criterion TGX (Tris-Glycine eXtended) Stain-Free 4%–20% gels. Gels were transferred to polyvinylidene fluoride membranes. After transferring, membranes were blocked with 5% skim milk for 1 h at room temperature. Then, membranes were incubated with the primary antibody (DCX or GluR1, see Table 1 for antibody concentrations) in a rocking plate overnight at 4 °C. The following day, membranes were washed with tris-buffered saline, 0.1% Tween and incubated with the appropriate secondary antibody for 1 h at room temperature. Membranes were visualised on an Amersham Imager 600 (GE Healthcare Life Sciences, Pittsburgh, PA, USA) using Luminata Classico western blotting detection reagent. The Amersham Imager 600 analysis software was (GE Healthcare Life Sciences, Pittsburgh, PA, USA) used to analyse the density of the bands.

### 4.11. Statistical Analyses 

All data were analysed using GraphPad Prism v7.02 (GraphPad Software, San Diego, CA, USA) and are expressed as mean ± standard deviation (SD). Tissue loss and motor test data were analysed using 2-way analysis of variance (ANOVA) followed by Sidak multiple comparisons. ELISA, western blotting and immunofluorescence labelling were analysed using 2-tailed t-tests. Pearson correlation was used to determine the association between motor performance and plasma IGF-1. A *p*-value < 0.05 was considered statistically significant.

## Figures and Tables

**Table 1:** List of antibodies used for western blot and immunofluorescence analyses.

Targets	Description	Sources of Antibodies	Application	Dilution
**BrdU**	Bromodeoxyuridine (BrdU) is used as a marker for the proliferation of cells [40]. It is a synthetic nucleoside analog of thymidine that is incorporated into the DNA of actively replicating cells.	Sigma-Aldrich, mouse anti-BrdU, #B8434	IF	1:1000
**NeuN**	Neuronal nuclei (NeuN) is a nuclear protein expressed in most neurons of the nervous systems [99]. It is a marker for mature neurons.	Cell Signalling, rabbit anti-NeuN (D3S31), #12943	IF	1:1000
**DCX**	Doublecortin (DCX) is a microtubule associated protein that stabilises and bundles microtubules. It is expressed by neuronal precursor cells and immature neurons [41]	abcam, rabbit anti-doublecortin, #ab18723	WB IF	1:1000 1:1000
**GluR1**	AMPA receptor 1 (GluR1) is an ionotropic glutamate-gated ion channel. GluR1 is implicated in synapse formation, stabilisation and plasticity [44]. GluR1 is necessary for expression of long-term potentiation in the hippocampus and formation of short-term memory [100].	Cell Signalling, rabbit anti-AMPA Receptor 1 (GluA1), #13185	WB IF	1:2000 1:1000
**β-actin**	β-actin is a cytoskeletal housekeeping protein.	Sigma-Aldrich, Monoclonal Anti-β-actin-HRP antibody, A3854	WB	1:50000
**NeuroTrace**	NeuroTrace fluorescent Nissl stain is selective for the Nissl substance present in neurons [101]. Nissl substance is composed of ribosomal RNA associated with the rough endoplasmic reticulum in neuronal perikarya and dendrites.	ThermoFisher Scientific, NeuroTrace™ 640/660 Deep-Red Fluorescent Nissl Stain, #N21483	IF	1:1000
**Lectin**	Tomato lectin is a common stain for blood vessels. Lectin binds to carbohydrate components of endothelial cells [80].	Vecton Laboratories, DyLight 649 Lycopersicon esculentum (Tomato) lectin #DL-1178	IF	1:1000
**Rabbit IgG**	Secondary antibody.	Biorad, Anti-Rabbit-HRP antibody, #170-6515	WB	1:7500
		ThermoFisher Scientific, anti-Rabbit IgG (H + L) Highly Cross-Adsorbed Secondary Antibody, Alexa Fluor 488, #A21206	IF	1:400
**Mouse IgG**	Secondary antibody.	ThermoFisher Scientific, anti-Mouse IgG (H + L) Highly Cross-Adsorbed Secondary Antibody, Alexa Fluor 594, #A21203	IF	1:400

WB, western blot; IF, immunofluorescence.

## Supplementary Materials

Supplementary Materials can be found at https://www.mdpi.com/1422-0067/21/2/606/s1.

## Abbreviations

GH	Growth hormone
rhGH	Recombinant human growth hormone
BrdU	Bromodeoxyuridine
IGF-1	Insulin-like growth factor 1
IGFBP-3	Insulin-like growth factor-binding protein 3
CL	Contralateral
IL	Ipsilateral
NeuN	Neuronal nuclei
DCX	Doublecortin
GluR1	AMPA Receptor 1
CD31	Cluster of differentiation 31
ELISA	Enzyme-linked immunosorbent assay
