# Supplementary material for: Growth Hormone Promotes Motor Function after Experimental Stroke and Enhances Recovery-Promoting Mechanisms within the Peri-Infarct Area
Source: Int J Mol Sci. 2020 Jan 17;21(2):606. doi: 10.3390/ijms21020606 (PMC7013985; doi:10.3390/ijms21020606)
Supplement: Supplementary file 1 [file ijms-21-00606-s001.pdf]

Supplementary Material

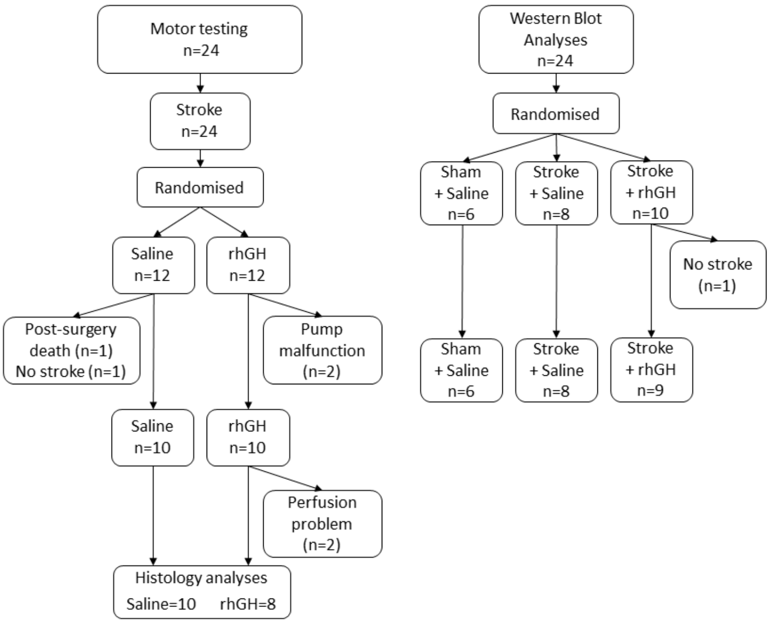

Supplementary Figure S1. Diagram for inclusion and exclusion of mice in this study. Mice were removed from the study if we histologically identified that the stroke had not occurred, or if malfunction of the mini-osmotic pumps had occurred. A total of 24 mice were used to assess motor function, and their brains were used for histological analysis (Stroke + Saline, *n* = 12; Stroke + rhGH, *n* = 12). A total of 24 sham mice were used for protein analysis of their brains (Sham + Saline, *n* = 6; Stroke + Saline, *n* = 8; Stroke + rhGH, *n* = 10).

Supplementary Material

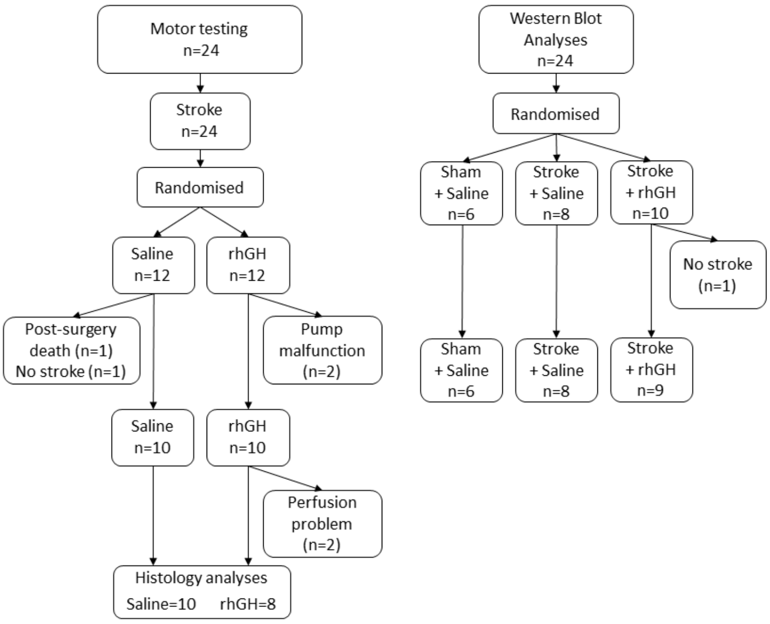

Supplementary Figure S1. Diagram for inclusion and exclusion of mice in this study. Mice were removed from the study if we histologically identified that the stroke had not occurred, or if malfunction of the mini-osmotic pumps had occurred. A total of 24 mice were used to assess motor function, and their brains were used for histological analysis (Stroke + Saline, *n* = 12; Stroke + rhGH, *n* = 12). A total of 24 sham mice were used for protein analysis of their brains (Sham + Saline, *n* = 6; Stroke + Saline, *n* = 8; Stroke + rhGH, *n* = 10).

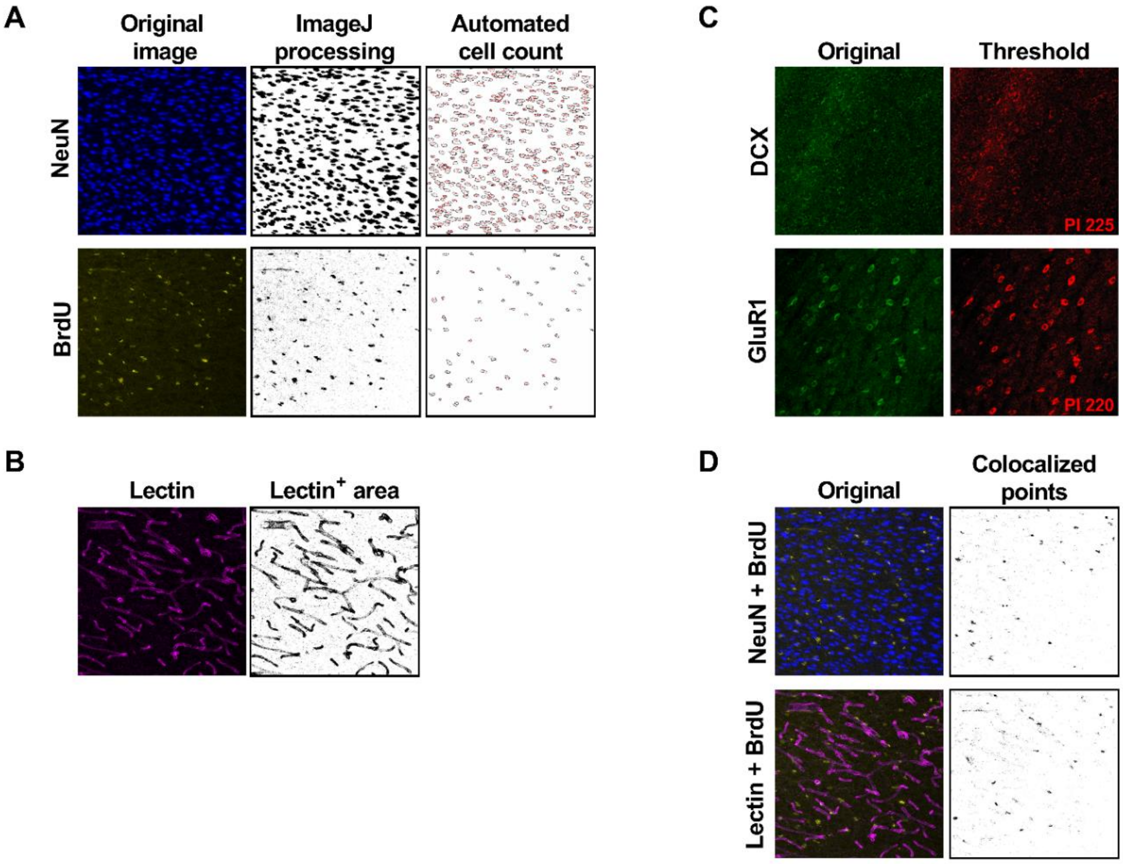

Supplementary Figure S2. Image processing and analysis. (**A**) Automated NeuN+ and BrdU+ cell counts were undertaken in the peri-infarct area using ImageJ software. The original images (left) were converted into a binary image (middle) and the black particles were counted (right). (**B**) To assess the area covered by vessels, we used lectin staining. Confocal images (left) were uniformly thresholded at a high stringency (right) using ImageJ. The Lectin+ area was expressed as a percentage of the overall field of view. (**C**) To analyse the DCX and GluR1 staining, we performed thresholding analyses and chose the optimal pixel intensity that clearly reflected the immunolabelled signal using ImageJ. The left panels represent the original images. The right panels show material thresholded at the pixel intensity (PI) 220 (DCX) and 225 (GluR1). The number of pixels that were captured at and below PI 125 were then expressed as a percentage of the total number of pixels in each image and this data was used to investigate group differences. (**D**) To assess the number of BrdU+ cells that have differentiated into neurons and blood vessels, we performed co-labelling analyses. Both channels (NeuN + BrdU or Lectin + BrdU) were merged together (left images). We then used the plugin 'colocalization' within ImageJ. This plugin highlights the colocalized points of two 8-bit images. The colocalized points appear in black (right images). The black particles were counted. Scale bar for all the images = 50μm.

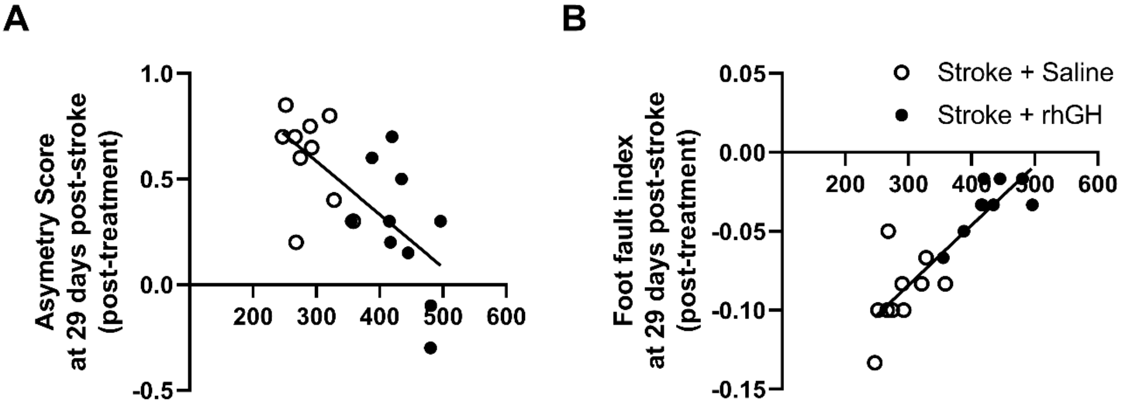

Supplementary Figure S3. Higher circulatory levels of IGF-1 are associated with better motor outcomes. (**A**) Pearson correlation analysis shows a significant positive correlation between serum IGF-1 levels and cylinder task performance post rhGH treatment ($r = -0.6789$; $p_{(Y = -0.002513X + 1.339)} = 0.0010$). (**B**) Pearson correlation analysis shows a significant correlation between serum IGF-1 levels and grid walk task performance post rhGH treatment ($r = 0.8879$; $p_{(Y = 0.0003785X - 0.1977)} < 0.0001$). x-axis represents plasma IGF-1 (ng/mL).
